# From reproductive technologies to genome editing in small ruminants: an embryo’s journey

**DOI:** 10.21451/1984-3143-AR2018-0022

**Published:** 2018-08-03

**Authors:** Alejo Menchaca, Pedro C. dos Santos-Neto, Frederico Cuadro, Marcela Souza-Neves, Martina Crispo

**Affiliations:** 1 Instituto de Reproducción Animal Uruguay (IRAUy), Montevideo, Uruguay; 2 Unidad de Animales Transgénicos y de Experimentación, Institut Pasteur de Montevideo, Uruguay

**Keywords:** cryopreservation, genome modification, IVF, MOET, ovine, transgenesis.

## Abstract

The beginning of this century has witnessed great advances in the understanding of ovarian physiology and embryo development, in the improvement of assisted reproductive technologies (ARTs), and in the arrival of the revolutionary genome editing technology through zygote manipulation. Particularly in sheep and goats, the current knowledge on follicular dynamics enables the design of novel strategies for ovarian control, enhancing artificial insemination and embryo production programs applied to genetic improvement. *In vitro* embryo production (IVEP) has evolved due to a better understanding of the processes that occur during oocyte maturation, fertilization and early embryo development. Moreover, interesting advances have been achieved in embryo and oocyte cryopreservation, thereby reducing the gap between the bench and on-farm application of IVEP technology. Nevertheless, the major breakthrough of this century has been the arrival of the CRISPR/Cas system for genome editing. By joining diverse disciplines such as molecular biology, genetic engineering and reproductive technologies, CRISPR allows the generation of knock-out and knock-in animals in a novel way never achieved before. The innumerable applications of this disruptive biotechnology are challenging the imagination of those who intend to build the animals of the future.

## Introduction

**Figure 1:**
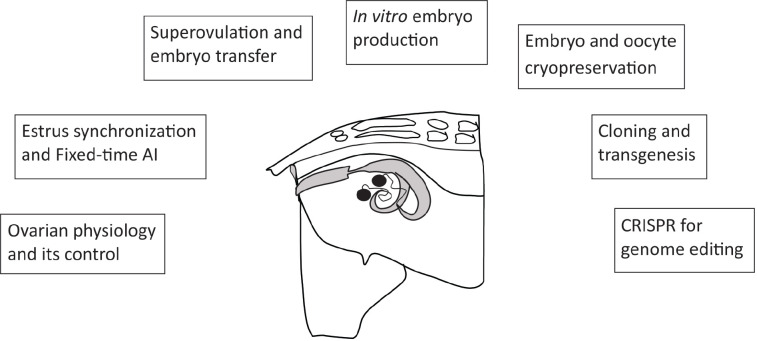
Contribution of the ewe/goat to the development of reproductive technologies. The understanding of ovarian physiology, as well as oocyte maturation, fertilization and embryo development, has allowed new advances in fixed-time artificial insemination (AI), in superovulation and in embryo transfer technologies. This has led to greater efficiency of *in vitro* embryo production and cryopreservation, application of modern technologies such as cloning and transgenesis, and more recently, embryo-related technologies have contributed with the arrival of the CRISPR/Cas system for genome editing.

Sheep and goats have been used in science not only because both species have great relevance as suppliers of food and wool/hair, but also due to their plasticity as experimental models for different purposes. Like Dolly - the world’s most famous sheep - these animals have been studied for basic reproductive physiology as well as for developing novel biotechnologies. In this review, we briefly describe the main advances of the last 20 years in both species related to ovarian physiology, the progress of reproductive technologies, and the contribution of embryo manipulation to genome editing (Fig. 1). Because extensive information has been discussed in previous reviews, we just highlight the latest advances and focus on the main results recently obtained in our laboratory.

## Follicular dynamics in sheep and goats

Since a deep knowledge of ovarian physiology is required for the control of reproduction and the application of assisted reproductive technologies (ARTs; Fig. 1), a brief update of ovarian follicular dynamics is presented. The follicular wave pattern in sheep and goats was clearly described in the 1990s with the advent of transrectal ultrasonography for the study of ovarian physiology (reviewed in sheep by Evans, 2003, and in goats by Rubianes and Menchaca, 2003). Follicular waves in these species have been reported during the estrous cycle, prepubertal period, seasonal anestrus and early gestation. This phenomenon is determined by the precise action of the endocrine system pathways through the combined action of gonadotropic hormones and steroids, as well as through the differential ability to express hormonal receptivity of the dominant or subordinate, growing or regressing, large or small follicles. The interrelationship between these endogenous factors has direct implications on the exogenous control of ovarian function for estrus synchronization and superovulation. During follicular wave emergence, the recruitment of small follicles is promoted by an FSH surge that precedes each wave, while after selection, the growth of medium and large follicles is supported by the LH hormone. Endogenous (and exogenous) progesterone influences follicular waves; high hormonal levels promote follicular turnover mainly by inhibition of LH support, while low progesterone levels promote the growth of the largest follicle, inducing a persistent follicle that negatively affects fertility. The emergence of each wave is unpredictable with the exception of wave 1, which emerges on day 0 in the interovulatory interval, soon after ovulation, and has practical implications for exogenous ovarian control. These mechanisms related to follicular waves pattern were extensively studied in several reports and are further described in previous reviews on sheep (Evans, 2003; Bartlewski *et al*., 2011) and goats (Rubianes and Menchaca, 2003).

Since follicular waves - especially follicular recruitment and dominance - have a substantial effect on the response to gonadotrophin and steroid administration, new hormonal protocols have been designed to improve pregnancy rates with a single insemination without the need for estrus detection (i.e., fixed-time artificial insemination or FTAI) or to enhance multiple ovulation and embryo transfer (MOET) programs.

## Synchronization of ovulation for FTAI

The information reported on follicular dynamics during the 1990s and 2000s was not considered in the traditional treatments for estrus synchronization, as they were designed in the 1970s- 1980s. The implementation of artificial insemination, particularly without estrous detection, requires a precise control of a) luteal function, b) follicular development, and c) ovulation. Traditional protocols were designed with the aim of controlling the luteal function by exogenous progesterone/progestogens administration for 10-14 days. The new protocols for FTAI achieve a better control of follicular development and ovulation that enhances fertility, mainly by reducing progesterone exposure from 10-14 days to 5-7 days (short-term protocols). This simple strategy avoids the detrimental effect of low progesterone concentrations during long periods when the intravaginal devices are placed for many days. These short-term protocols for FTAI (Menchaca and Rubianes, 2004) consist of exposure to exogenous progesterone (usually in a CIDR-type intravaginal device) for 5-7 days, associated with a dose of equine chorionic gonadotropin (eCG) and prostaglandin (PG) F2α at the time of device removal. This protocol induces high progesterone concentrations that promote follicular turnover soon after device insertion (low LH support), and leads to the growth of a new follicle that reaches a preovulatory diameter 5-7 days after the intravaginal device insertion. Estrus, LH peak and ovulation occur approximately 30, 40 and 60 h after device removal, respectively (goats: Menchaca *et al*., 2007a; Vilariño *et al*., 2011; sheep: Vilariño *et al*., 2010, 2013). The pregnancy rate obtained with the short-term protocol associated with FTAI, and associated with natural mating or conventional artificial insemination, has been previously published in several reports in sheep and goats (Ungerfeld and Rubianes, 1999; Menchaca and Rubianes 2004, 2007; Fonseca *et al*., 2005, 2017). In addition, we have recently generated new information on large-scale FTAI programs on more than 13,000 ewes (Menchaca, 2018; IRAUy, Montevideo, Uruguay; unpublished results). In these programs, progesterone priming was administered by using intravaginal devices containing 0.3 g of progesterone (DICO, Syntex, Argentina) as described previously (Vilariño *et al*., 2010; Santos-Neto *et al*., 2015a). In one experiment, the short-term (6 days) *vs.* long-term (14 days) protocol was compared in 1,750 multiparous sheep that received intrauterine insemination by laparoscopy. The pregnancy rate was significantly higher with the short-term rather than the long-term treatment (43.5 *vs.* 37.8%, respectively; P < 0.05). In a following experiment, to further compare high *vs.* low progesterone concentrations, 922 females were treated for 6 days with a new intravaginal device (high progesterone for a short time) or for 14 days with a second-use device previously used for 6 days (low progesterone for an extended length). The pregnancy rate was also higher for the shorter treatment (41.2 *vs.* 29.1%, respectively, P < 0.05). These results confirm previous studies reported in sheep and goats (Menchaca and Rubianes, 2004), adding more evidence to the concept that fertility falls as the progesterone levels decreases when using intravaginal devices for long periods.

**Figure 2:**
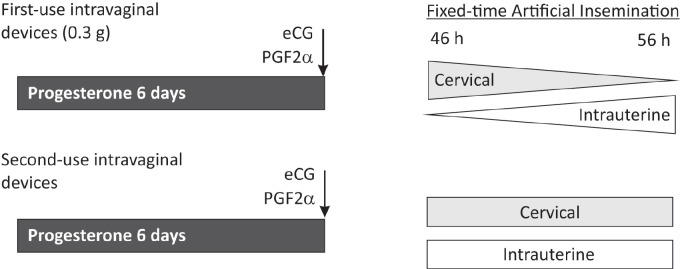
The short-term protocol for Fixed-time Artificial Insemination (FTAI) in sheep and goats. The protocol consists of progesterone treatment, administered by intravaginal devices (e.g., CIDR or DICO) for 5-7 days associated with one dose of equine chorionic gonadotrophin (eCG) and prostaglandin (PG) F2α at the time of device removal. First-use (new devices) or second-use (used previously by 5-7 days) intravaginal devices can be used. In sheep, for first-use devices, FTAI should be performed on the morning of day 8 (46-50 h after device removal) by the cervical route, or in the afternoon (52-56 h) by the intrauterine route. For second-use devices, FTAI could be performed by both insemination routes in the morning or in the afternoon without affecting fertility.

In another experiment on 3,893 multiparous ewes, we evaluated the best moment for FTAI with this 6-day protocol followed by cervical or intrauterine insemination from 46 to 56 h after device removal (the progesterone device was removed in the morning on day 6; Menchaca, 2018; IRAUy, Montevideo, Uruguay; unpublished results). When new devices were used, the greatest pregnancy rate with cervical insemination was obtained when FTAI was performed on the morning of day 8 (i.e., 46 to 50 h from device removal) rather than in the afternoon (i.e., 52 to 56 h), while with intrauterine insemination greater pregnancy rate was obtained with FTAI in the afternoon. Interestingly, the pregnancy rate with second-use devices was similar between those ewes with FTAI in the morning and in the afternoon, both by cervical and intrauterine insemination. This difference between new and used devices is probably related to a wide period of ovulation in the females treated with used devices (Fig. 2). The Short-term protocol for FTAI in sheep has also been evaluated by transcervical insemination route through cervical retraction, achieving an intermediate pregnancy rate between conventional cervical and laparoscopic intrauterine insemination (Casali *et al*., 2017).

In summary, different studies reported during the last few years show that short-term protocols using intravaginal progesterone devices result in a series of benefits compared with the long protocols used previously, namely, better control of follicular response and ovulation, acceptable pregnancy rates, shorter periods for implementation, and eventually, the possibility of reuse of silicone devices, thus reducing the cost of the treatment. The current protocol for FTAI applied in our practice is depicted in Fig. 2.

## Superovulation and *in vivo* embryo production

**Figure 3:**
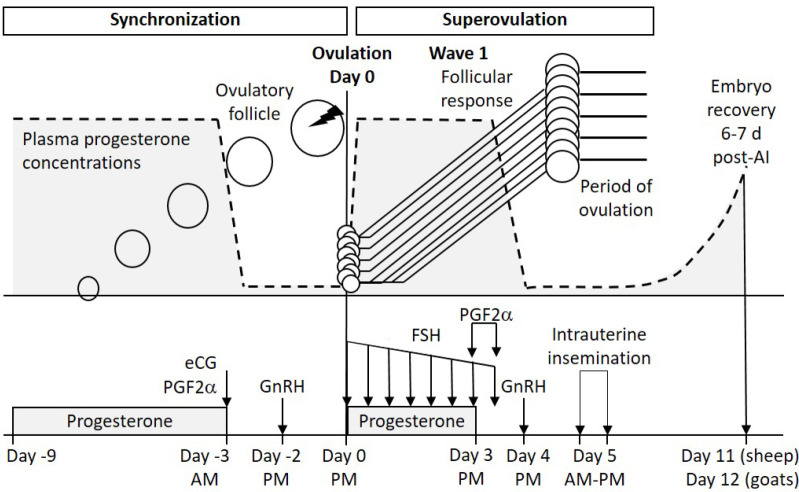
Ovarian superstimulatory treatment (Day 0 protocol) for embryo production in sheep and goats (adapted from Menchaca et al., 2010; Cuadro et al., 2018). During the synchronization period (left), the ovulation is induced to synchronize the emergence of wave 1. During the superovulation period (right), the FSH is administered to promote follicular recruitment of wave 1 in absence of a large dominant follicle. Additionally, progesterone treatment using an intravaginal device (i.e., CIDR-type device) is given during FSH administration to induce greater progesterone levels (gray color under dotted line) during superstimulated follicular growth. Prostaglandin (PG) F2α is administered in two half doses 12 h apart (the first one is given at device removal), GnRH is given 24 h later, and intrauterine insemination is performed 16-24 h after GnRH. Uterine flushing is performed 6 and 7 days after insemination in sheep and goats.

The current knowledge about follicular dynamics has also enabled the development of new superstimulatory treatments for embryo production. It has been shown that the presence of a dominant follicle at the beginning of a superstimulatory treatment has a detrimental effect on the response to superovulation and embryo production (reviewed by Menchaca *et al*., 2010). Because 70 to 85% of donors have a dominant follicle at the moment of the first FSH administration in conventional treatments (Veiga-Lopez *et al*., 2005; Menchaca *et al*., 2007b), at least three new alternatives to synchronize the emergence of a new follicular wave before FSH administration have been proposed by different authors (Menchaca *et al*., 2002, 2007b; Cognié *et al*., 2003; Bartlewski *et al*., 2008). In general, these three strategies results in a better control of follicular dynamics and a greater superovulatory response, taking advantage of the spontaneous recruitment that normally occurs within the emergence of a follicular wave. One of these treatments is known as the Day 0 protocol, which consists of the superstimulation of wave 1 (Menchaca *et al*., 2002, 2007b, 2009, 2010). This protocol initiates FSH treatment when the first follicular wave emerges at the time of ovulation (i.e., on day 0 of the cycle), thus requires the synchronization of the ovulation of the dominant follicle to promote follicular turnover and the emergence of wave 1. The Day 0 protocol is depicted in Fig. 3. This treatment has improved ovarian response and embryo production compared to traditional treatments, both in sheep and goats (Menchaca *et al*., 2010).

We have recently demonstrated in sheep the convenience of exposing the oocyte to high progesterone concentrations prior to maturation, i.e., during preovulatory follicular development (Cuadro *et al*., 2018; IRAUy, Montevideo, Uruguay; submitted article). Interestingly, the induction of high progesterone levels for three days before luteolysis (i.e., during the FSH treatment) improves fertilization rate and embryo yield. In a subsequent study in which the oocytes were aspirated and subjected to *in vitro* fertilization, it was demonstrated that this enhancement was due to a greater oocyte developmental competence (Menchaca *et al*., 2018). For this reason, we have added into the Day 0 protocol the administration of an intravaginal device with progesterone during the FSH treatment (Fig. 3). Because the use of progestogens instead of progesterone does not always induce the same response (Santos Neto *et al*., 2015a), the use of progestogens should be evaluated before its application in this superstimulatory treatment. These and other refinements have been incorporated in the Day 0 protocol by different authors (Tasdemir *et al*., 2011; Balaro *et al*., 2016; Lima *et al*., 2016; Mogase *et al*., 2016; Souza-Fabjan *et al*., 2017).

Before these new approaches for superovulation were developed through the control of follicular waves, some improvements to the traditional protocols had been proposed. In a large-scale program with 4,262 produced sheep embryos (Menchaca *et al.*, 2009), we attempted to enhance traditional protocols consisting of 12-14 days of exposure to progesterone before FSH administration. In one of these experiments on 239 donor sheep, the length of progesterone priming was evaluated to determine whether 12-14 days of exposure was necessary. Ewes were exposed to progesterone using CIDR-G (0.3 g of progesterone, Zoetis) for 5, 6, 7, 8, 9, 10, 11, 12, 13 or 14 days (23 to 25 donors in each experimental group). The results showed no significant differences in any of the evaluated variables with a similar embryo production, concluding that the length of the treatment could be more flexible, which has practical advantages for application in large-scale programs (Menchaca *et al*., 2009). In another experiment, the use of eCG associated with the administration of FSH was evaluated on 264 donor ewes. This has interesting implications because, even though no clear evidence was available, many practitioners use one dose of eCG at device removal during the FSH treatment, assuming that its LH action could promote final follicular development, enhancing embryo production. However, in this study, the treatment with eCG reduced the quantity and quality of produced embryos, suggesting the elimination of eCG at the end of FSH administration (Menchaca *et al*., 2009). In another experiment, in order to improve the synchronization of the ovulation and improve the embryo yield, the effect of GnRH administration 24 hours after CIDR removal was evaluated on 161 donor ewes. The GnRH treatment increased the fertilization rate and enhanced embryo production, and thus, we recommend the systematic use of GnRH after FSH administration (Menchaca *et al*., 2009). In summary, although traditional protocols have shown certain weaknesses that can affect the quantity and quality of the produced embryos (compared to the protocol for wave 1), for those practitioners that still use these treatments, the adjustments described above are recommended.

Superovulation and embryo production is a well-known technology, and for this reason, many other intrinsic and extrinsic factors affecting its success have been clearly identified. Since not all of the published information can be included, for further information we recommend previous reviews encompassing more global ideas of this technology (Gonzalez Bulnes *et al*., 2004; Menchaca *et al*., 2010; Bartlewski *et al*., 2016).

## *In vitro* embryo production

Significant fine-tuning of *in vitro* embryo production (IVEP) technology has been achieved from a better understanding of different molecular and biochemical events that occur during oocyte maturation, fertilization and early embryo development. In addition to the advantages of *in vivo* embryo production related to selective breeding, the *in vitro* system in livestock also allows the production of offspring from females that would not be able to reproduce using artificial insemination or MOET, such as prepubertal animals. The IVEP system is also useful for species conservation programs, and represents a valuable research tool in developmental biology and in the study of human infertility treatments. Even more interestingly, this technology provides the platform for the implementation of other technologies such as cloning, transgenesis and genome editing.

The success of an IVEP program depends largely on the availability of a continuous number of good quality oocytes. Although slaughterhouses represent a low-cost and abundant source of oocytes useful for research projects, oocytes from live animals are required for commercial application of IVEP. For this purpose, follicular aspiration by laparoscopy (LOPU) is mandatory in sheep and goats, providing approximately 10-14 oocytes per female in each session (Baldassarre *et al*., 2002, 2003b; Teixeira *et al*., 2011). Follicular aspiration of live animals needs to be associated with ovarian stimulation, usually achieved by using a single dose of FSH and eCG 36 h before LOPU (Baldassarre *et al*., 1996; Gibbons *et al*., 2007). The control of follicular dynamics previous to aspiration to improve *in vitro* oocyte developmental competence has been recently proposed (Menchaca *et al*., 2018) and further investigations are required.

Once cumulus oocyte complexes (COCs) are obtained, the success of the following steps depends, in addition to oocyte quality, also on the *in vitro* culture environment. Thus, culture media composition and protocols are determining factors for *in vitro* maturation (IVM), fertilization (IVF) and culture (IVC), having a direct impact on pregnancy rate and some long-term consequences on offspring traits (Thompson *et al*., 2007). There are different *in vitro* media systems proposed and adopted, some of which are made in the laboratory and some of which are commercially available. The most commonly used medium for IVM in sheep and goats is tissue culture medium (TCM199) supplemented with estrus sheep/goat serum, gonadotrophins, cysteamine and antibiotics. For IVF, usually synthetic oviduct fluid (SOF) supplemented with heparin, hypotaurine and estrus sheep serum is used. After fertilization, the recommended IVC system in general consists of serum-free media under defined or semi-defined conditions, sequential or not, and always designed to suit embryo requirements. The embryo culture media and procedures most likely differ between laboratories, which also represents a source of variation. The main features of the procedures used in our laboratory are available in detail in previous reports (see Menchaca *et al*., 2016b). Under these conditions, the expected cleavage rate is approximately 80-90%, and the blastocyst rate is approximately 30 to 40% (number of blastocysts on day 6 from COCs in IVF). For further information about other factors affecting the success of IVEP, see recent reviews by Souza-Fabjan *et al*. (2014), Paramio and Izquierdo (2016) and Menchaca *et al*. (2016b).

## Embryo cryopreservation

Embryo cryopreservation in sheep and goats was first reported in the 1970s by the slow freezing method for *in vivo* derived embryos, which has received moderate improvements during the recent years. On the other hand, novel information has been published with vitrification by minimum volume methods, mainly focusing on the cryotolerance of *in vitro* produced embryos.

Slow freezing is the default method for *in vivo* derived embryos used by many practitioners worldwide, resulting in good embryo cryotolerance and acceptable pregnancy rates. However, when slow freezing is applied to *in vitro* produced embryos, low outcomes are achieved (Massip, 2001, Santos-Neto *et al*, 2017). Substantial efforts and some interesting strategies have been proposed to improve the survival rate of *in vitro* produced embryos subjected to slow freezing, mainly in bovine embryos (Sudano *et al*., 2013; Sanches *et al*., 2016). However, the application of slow freezing to IVEP programs remains controversial. Multiple factors are associated with the lower cryotolerance of embryos produced *in vitro* compared with embryos produced *in vivo* (Seidel, 2006), such as excessive cytoplasmic lipid content, changes in the structural, physic and chemical characteristics of the embryo, the stage of embryo development, media composition, and protocols. Usually, to avoid the low embryo survival after cryopreservation, IVEP programs are conducted with fresh embryos. For this type of programs (IVEP with fresh embryos) in large-scale operations with many embryos being produced every week during long periods, requires a large number of ready-to-use recipients. In addition, all the well-known advantages of cryopreservation related to international trade and genetics dissemination remain limited for IVEP technology. In this context, new approaches for embryo cryopreservation deserve to be considered.

**Figure 4:**
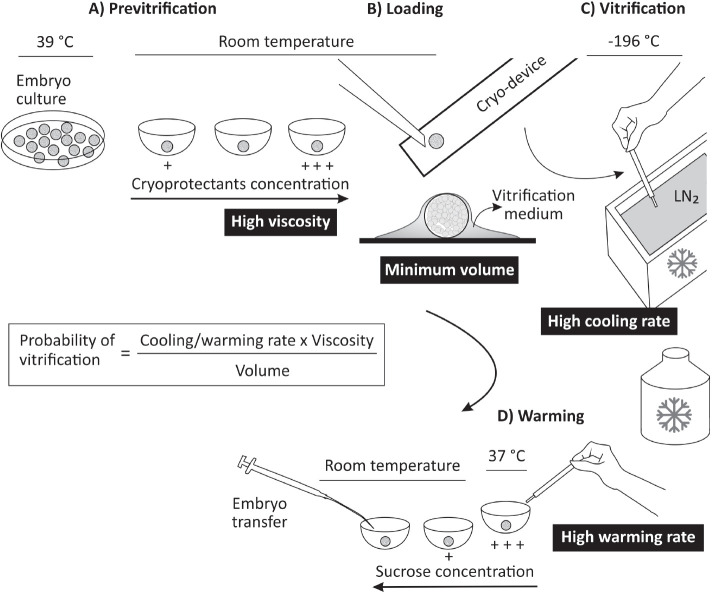
Embryo cryopreservation by minimum volume vitrification (e.g., Cryotop or Spatula) used in our Laboratory (dos Santos et al., 2015; 2017; Meikle et al., 2018; Barrera et al., 2018). Before vitrification (A), the embryos are equilibrated at room temperature with increasing concentrations of cryoprotectants to reach high viscosity. The embryos are loaded on a cryo-device (Cryotop or Spatula) (B), and the vitrification media is removed from the surface until reaching to reach minimum volume (i.e., ~ 1 µl). Then, the loaded device is rapidly plugged into liquid nitrogen (LN_2_) to reach an ultra-fast cooling rate (C). Warming is performed by plugging the device directly from LN_2_ into a sucrose containing media at 37°C (D). Embryos are washed successively in decreasing sucrose concentrations and handling medium, and finally are transferred into recipient females. This protocol sticks to the concept proposed by Arav (2014) to enhance the probability of vitrification (i.e., high cooling/warming rate, high viscosity, minimum volume). The methods have been modified from Kuwayama (2007) and Tsang and Chow (2009).

Since the 1990s, several methods of vitrification have been proposed in small ruminants as an alternative to slow freezing, both for *in vivo* derived and *in vitro* produced embryos. Vitrification has been reported in these species with different success rates, in reports comparing different types of cryoprotectants and times of exposure, cryo-devices and protocols (Traldi *et al*., 1999; Dattena *et al*., 2000; Papadopoulos *et al*., 2002; Cognié *et al*., 2003; Martínez *et al*., 2006;

Gibbons *et al*., 2011; Ferreira-Silva *et al*., 2017). More recently, the novel concept of minimum volume vitrification, with ultra-high cooling rates and high media viscosity, has appeared as a renewed hope for progress in embryo cryopreservation in various species (Arav, 2014). This idea has also been evaluated in caprine (Morató *et al*., 2011) and ovine embryos (Santos-Neto *et al*., 2015b; 2017) with promising results. We have been conducting a series of experiments with ovine embryos using the new minimum volume vitrification methods Cryotop and Spatula MVD. Both vitrification methods were reported for the first time in humans (Kuwayama, 2007) and mice embryos (Tsang and Chow, 2009), respectively and are routinely used in our laboratory for ovine (Santos-Neto *et al*., 2015b; 2017) and murine embryos (Meikle *et al*., 2018; Institut Pasteur, Montevideo, Uruguay, submitted article). In brief, the ovine IVP embryos vitrified with both minimum volume methods at different stages (at 2 and 6 days after IVF) showed acceptable *in vitro* survival, development and hatching rates (Santos-Neto *et al*., 2015b). In a subsequent study (Santos-Neto *et al*., 2017), we compared the pregnancy outcomes of 437 *in vivo* derived and *in vitro* produced embryos submitted to vitrification by the Cryotop or the Spatula MVD methods, or submitted to conventional freezing. Interestingly, the pregnancy rate after fixed-time embryo transfer was significantly greater for the Cryotop method, both for *in vivo* and *in vitro* embryos. For *in vivo*-derived embryos, vitrification by Cryotop reached a remarkable pregnancy rate of 67.1% (pregnant/transferred embryos), while for slow freezing it was 45.6% (P < 0.05) that is considered normal for frozen embryos. For *in vitro-*produced embryos, the pregnancy rate was 55.1% and 7.3% for Cryotop and conventional freezing, respectively (P < 0.05), which confirms the extremely low outcomes with slow freezing and demonstrates the acceptable performance with the Cryotop method. The results of the Spatula MVD method were intermediate (Santos-Neto *et al*., 2017). Therefore, vitrification by minimum volume methods appears to be an interesting cryopreservation tool for future implementation of IVEP programs, at least in sheep, and may be an alternative for replacement of slow freezing technology in conventional MOET programs. Vitrification by minimum volume methods is depicted in Fig. 4.

Recently, we have included the minimum volume method Cryotop in the routine production of microinjected embryos for CRISPR gene editing technology. The possibility of temporary dissociation among embryo production and transfer, simplifies the complex operation of carrying out IVEP, micromanipulation protocols for CRISPR injection, recipient management, estrous synchronization, and embryo transfer simultaneously. This is particularly important in this kind of large-scale projects. Additionally, regarding the relevance of oocyte cryopreservation for different species (Vajta, 2000; Ledda *et al*., 2007), vitrification by minimum volume methods has been evaluated also in sheep oocytes with promising results (Succu *et al*., 2008; Barrera *et al*., 2018). However, even though important advances have been achieved on cryopreservation of *in vitro* produced embryos and oocytes, further investigations and some refinements are still necessary in order to obtain an easy, fast, low-cost and effective method for a wider application of this ART.

## Embryo micromanipulation for genetic modification

Cell plasticity in terms of totipotency and pluripotency in zygotes and early embryos, respectively, enables novel strategies for genetic manipulation in experimental and farm animals. Traditionally, the ovine and caprine zygote has been microinjected into the pronucleus in order to add exogenous DNA to obtain transgenic founders for a specific gene (reviewed by Menchaca *et al*., 2016a). In these species, the pronucleus is difficult to visualize mainly because they contain a huge quantity of lipid droplets inside the cytoplasm, and centrifugation is usually required. The injection pipette loaded with the DNA fragment is inserted into the pronucleus and the DNA is released inside until swelling occurs. Although pronuclear microinjection was the unique technique for genome modification for many years, it has some disadvantages in livestock: i.e., <10% transgenic offspring efficiency, unpredictable gene integration and expression, high cost and time consuming projects, eventually with low feasibility and sometimes with frustrating results (Menchaca *et al*., 2016a). Some technical difficulties were overcome by other available techniques, such as SCNT or lentiviral vectors. We have reported interesting results with transgenesis mediated by lentivirus in sheep (100% gene integration and 88.9% of expression in 9 lambs produced with GFP reporter gene), showing some additional advantages such as high efficiency through perivitelline microinjection of zygotes or 2-cell embryos instead of pronuclear microinjection or nuclear transfer (Crispo *et al*., 2015b). Transposons system (Bevacqua *et al*., 2017) and sperm-mediated gene transfer (Pereyra-Bonnet *et al*., 2011) have been reported in sheep or goats although scarce information is available in these species.

Since Dolly (Wilmut *et al*., 1997), and particularly since Polly and Molly (Schnieke *et al*., 1997), SCNT has been the default method for generating transgenic farm animals. The procedure has been extensively described in small ruminants (Keefer *et al*., 2001; Campbell, 2002; Menchaca *et al*., 2016a), and although some laboratories use this tool as routine, the technique is laborious and time consuming, with low final efficiency and fetal/placenta problems or newborn alterations (Martins *et al*., 2016). In sheep, some reports describe the use of this technique to generate transgenic animals expressing or overexpressing exogenous or endogenous genes, respectively (Schnieke *et al*., 1997; Deng *et al*., 2013; Zhang *et al*., 2013), or more recently, knock-out models combining the use of SCNT with cells edited through the use of endonucleases (Li *et al*., 2016). More information can be found in goats, with several reports describing a wide diversity of interesting models (e.g., Baguisi *et al*., 1999; Baldassarre *et al*., 2003a; Yu *et al*., 2012).

Recently described, the CRISPR/Cas (clustered regulatory interspaced short palindromic repeats/CRISPR associated protein) system allows the microinjection of single guides of RNA (sgRNA) directly into the cytoplasm, with no need to centrifuge the zygote as in pronuclear injection, or avoiding the nuclear reprogramming required for SCNT. In addition, it shows a high embryo survival and pregnancy rate with uncommon fetal and offspring losses, as well as milder ethical concerns. Most impressively, this tool allows not only add new DNA as the previous aforementioned tools, but also to silence or correct endogenous genes, or to introduce mutations in the genome in a way never achieved before. This biotechnology was selected as Science’s 2015 Breakthrough of the Year (Travis, 2015) and represent one of the great advances, if not the greatest, of this century in biology and related fields.

## CRISPR/Cas for genome editing

The first knock-out (KO) animal model produced with the CRISPR/Cas system was reported in mice in 2013 (Wang *et al*., 2013). Subsequently, new births were achieved in other species including sheep (Crispo *et al*., 201*5a*), goats (Wang *et al*., 2015), and more recently, cattle (Gao *et al*., 2017). CRISPR is the third generation of restriction endonucleases (enzymes with the ability to cut specific regions of DNA), and it has been proven to be much more efficient and easy to apply than its predecessors, namely, zinc finger nucleases (ZFN) and transcription activator-like effector nucleases (TALEN). The uniqueness of CRISPR/Cas lies in the use of RNA instead of proteins to confer target specificity. The different components of the system, including CRISPR RNA (crRNA), trans- activating crRNA (tracrRNA) and the Cas9 enzyme, work together to make the process effective and efficient. Basically, these molecular scissors recognize and bind a specific DNA sequence, producing double strand breaks that can be repaired by the host DNA repair mechanisms. This repairing can be done by non-homologous end joining (NHEJ) or by homology-directed repair (HDR) in order to produce insertions or deletions, which can cause a frameshift mutation and thus a null allele, or the exchange of a few nucleotides or even transgene insertion if the repair is through HDR. Due to its high efficiency, its easy and fast laboratory setup, and its unlimited number of applications, the CRISPR/Cas system is a real revolution in several disciplines.

**Figure 5:**
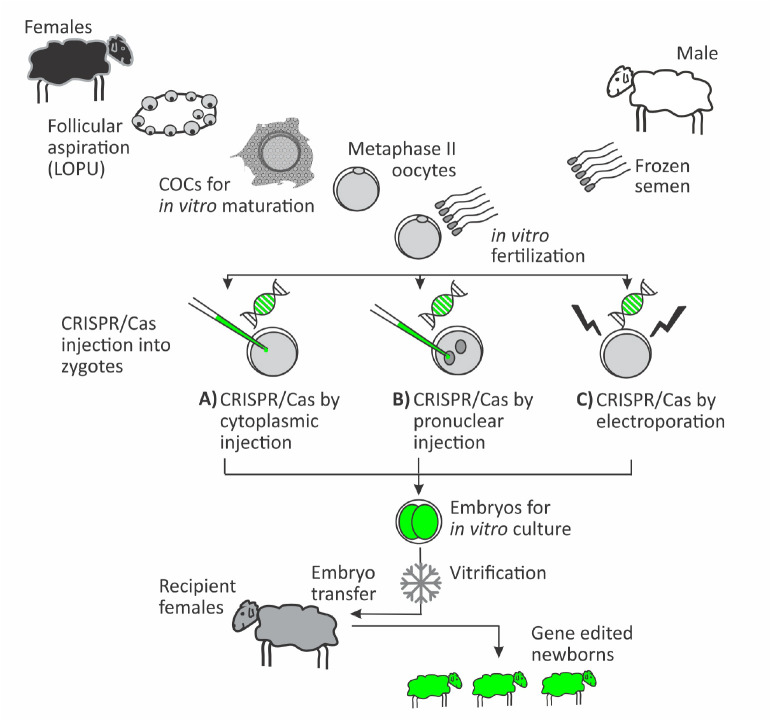
Schematic representation of embryo production and CRISPR microinjection for the generation of genome edited sheep and goats (adapted from Menchaca et al., 2016b). Immature oocytes can be obtained from live animals by Laparoscopic Ovum Pick-Up (LOPU) or from slaughterhouse ovaries. After *in vitro* maturation and fertilization, zygotes are prepared for CRISPR/Cas injection. The CRISPR delivery is performed by cytoplasmic microinjection (the default method in farm animals) (A), pronuclear microinjection (used in mice and rats) (B), or electroporation without the need of embryo micromanipulation (reported only in rats and mice) (C). Microinjected embryos are maintained in *in vitro* culture until fresh transfer or vitrification.

CRISPR technology applied in ruminants could be designed to enhance meat and wool production, increase the yield and quality of milk, generate disease resistant animals, provide animal resilience to hostile environments and enhance animal welfare, or to reproduce human diseases for biomedicine application. There is worldwide scientific consensus that this technology is far better than the previous tools. The relatively simply molecular biology setup, the ease of zygote microinjection into the cytoplasm, and the high mutation rate efficiency makes this tool available to many more laboratories working in different species. The sgRNA and Cas (RNA or protein) are mixed and loaded into the microinjection pipette, and few picoliters are injected into the cytoplasm or pronucleus of zygotes soon after fertilization. Surviving zygotes are left in culture until embryo analysis, cryopreservation or transfer (Fig. 5). In these conditions, acceptable outcomes have been obtained in sheep and goats with CRISPR injection into zygotes (Menchaca *et al*., 2016a). At the end of 2014, our first lambs edited by CRISPR/Cas9 were born (Crispo *et al*., 2015a) in a KO model to disrupt the myostatin gene (a gene encoding for a protein that inhibits muscle growth). We obtained 45.4% born lambs showing mutations at the myostatin locus, resulting in a body weight increase of 25% when compared to wild type counterparts. In a more recent sheep model of disease resistant animals (Menchaca, 2018; IRAUy, Montevideo, Uruguay; unpublished results), we obtained a 53.8% mutation rate by NHEJ in preimplantation embryos; additionally, in a following project to produce a human disease sheep model, we obtained 50.0% mutant embryos confirming the efficiency of this technique. Currently, about ten models for each species (sheep and goats) have reported successful births and several projects are ongoing in different laboratories worldwide. This technology is more recent in cattle, with the first birth of CRISPR-edited calves reported last year in China (Gao *et al*., 2017). These extraordinary outcomes and acceptable efficiencies encourage the widespread use of CRISPR/Cas to generate large animal models, including knock-outs and knock-ins with different purposes.

In order to refine and the CRISPR technology, different improvements are continuously being published. Reducing off-target sites by the use of nickases (Frock *et al*., 2015), improving HDR using SCR7 as an inhibitor of NHEJ (Vartak and Raghavan, 2015) or using Cas9 variants, Cas9 homologs and novel Cas proteins other than Cas9 (Nakade *et al*., 2017), will allow improvement of the efficiency and specificity of our models, including the targeting of multiple gene loci, generating knock-down or knock-in models, or include fluorescence imaging. Another improvement in overall efficiency is the introduction of CRISPR/Cas9 to zygotes through electroporation (Hashimoto *et al*., 2016; Remy *et al*., 2017), avoiding the use of expensive equipment and high skill human resources, with the possibility to edit hundreds of zygotes in few minutes. These and other improvements are envisioned for the following years, enhancing even more the power of this novel biotechnology.

## Final remarks

Better understanding of ovarian physiology and embryo development has allowed the progress of artificial insemination and embryo transfer technology, mainly applied to genetic improvement and breeding programs. The *in vitro* technology for oocyte maturation, fertilization and embryo development open new opportunities for genetic improvement and, more importantly, for the development of innovative biotechnologies through embryo micromanipulation. Cryopreservation of *in vitro* produced embryos in small ruminants has been advanced recently, but further refinements are required. These advances have been useful but slightly modest, and they have mainly focused on the improvement and not on the disruption of existing technologies. On the other hand, genome editing appears to be a novel and powerful approach, and in contrast to previous breakthrough technologies that can take years of experience to master, the CRISPR system enables the rapid and widespread application of genome editing by new users in almost every species. In the coming years, this technology will be applied in livestock through the support of genetics industry, in public health through biomedical businesses, and in basic and applied research conducted by different scientific organizations. The animals of the future will be different, the CRISPR revolution has only just begun.
